# Functional characterization reveals that zebrafish CFTR prefers to occupy closed channel conformations

**DOI:** 10.1371/journal.pone.0209862

**Published:** 2018-12-31

**Authors:** Jingyao Zhang, Ying-Chun Yu, Jiunn-Tyng Yeh, Tzyh-Chang Hwang

**Affiliations:** 1 Department of Bioengineering, University of Missouri-Columbia, Columbia, Missouri, United States of America; 2 Dalton Cardiovascular Research Center, University of Missouri-Columbia, Columbia, Missouri, United States of America; 3 Department of Medical Pharmacology and Physiology, University of Missouri-Columbia, Columbia, Missouri, United States of America; Xuzhou Medical University, CHINA

## Abstract

Cystic fibrosis transmembrane conductance regulator (CFTR), the culprit behind the genetic disease cystic fibrosis (CF), is a phosphorylation-activated, but ATP-gated anion channel. Studies of human CFTR over the past two decades have provided an in-depth understanding of how CFTR works as an ion channel despite its structural resemblance to ABC transporters. Recently-solved cryo-EM structures of unphosphorylated human and zebrafish CFTR (hCFTR and zCFTR), as well as phosphorylated ATP-bound zebrafish and human CFTR offer an unprecedented opportunity to understand CFTR’s function at a molecular level. Interestingly, despite millions of years of phylogenetic distance between human and zebrafish, the structures of zCFTR and hCFTR exhibit remarkable similarities. In the current study, we characterized biophysical and pharmacological properties of zCFTR with the patch-clamp technique, and showed surprisingly very different functional properties between these two orthologs. First, while hCFTR has a single-channel conductance of 8.4 pS with a linear I-V curve, zCFTR shows an inwardly-rectified I-V relationship with a single-channel conductance of ~3.5 pS. Second, single-channel gating behaviors of phosphorylated zCFTR are very different from those of hCFTR, featuring a very low open probability *P*_*o*_ (0.03 ± 0.02, vs. ~0.50 for hCFTR) with exceedingly long closed events and brief openings. In addition, unlike hCFTR where each open burst is clearly defined with rare short-lived flickery closures, the open bursts of zCFTR are not easily resolved. Third, although abolishing ATP hydrolysis by replacing the catalytic glutamate with glutamine (i.e., E1372Q) drastically prolongs the open bursts defined by the macroscopic relaxation analysis in zCFTR, the *P*_*o*_ within a “locked-open” burst of E1372Q-zCFTR is only ~ 0.35 (vs. *P*_*o*_ > 0.94 in E1371Q-hCFTR). Collectively, our data not only provide a reasonable explanation for the unexpected closed-state structure of phosphorylated E1372Q-zCFTR with a canonical ATP-bound dimer of the nucleotide binding domains (NBDs), but also implicate significant structural and functional differences between these two evolutionarily distant orthologs.

## Introduction

As a major breakthrough in cystic fibrosis (CF) research, three high-resolution cryo-EM structures of cystic fibrosis transmembrane conductance regulator CFTR (unphosphorylated human and zebrafish CFTR, and phosphorylated ATP-bound zebrafish and human CFTR) were published recently [1–4]. These structures have provided not only unprecedented insights for the interpretations of functional data accumulated over the years, but also exquisite guides for future structural and functional interrogations of CFTR [5]. Consistent with the basic architecture of CFTR proposed since the identification of the *cftr* gene [6], all four structures show two Transmembrane Domain (TMD)—Nucleotide Binding Domain (NBD) complexes, linked by a disordered Regulatory Domain (RD) mostly unresolved with the cryo-EM technique. These structures support a model established previously for CFTR’s function as an ion channel [7–11]: After multiple serines/threonines in the RD are phosphorylated by Protein Kinase A (PKA), ATP molecules, serving as a molecular glue, join two NBDs together to form a tight head-to-tail NBD dimer. This NBD dimerization is coupled to the opening of the gate in the TMDs [12]. Then, ATP hydrolysis and the subsequent release of hydrolytic products from the ATP binding pockets trigger channel closure ([13], and cf. [14, 15]). Specifically, the unphosphorylated apo-forms of human and zebrafish CFTR (hCFTR and zCFTR respectively) show two widely separated NBDs and an anion permeation pathway crafted by the TMDs but closed at the external end [1, 4]. Detailed comparisons of the structures of these two CFTR orthologs show remarkable similarities at a molecular level, despite a far-flung evolutionary distance and divergent living environments between these two species [16, 17]. Moreover, the phosphorylated, ATP-bound structure of zebrafish and human CFTR show a head-to-tail NBD dimer with two ATP molecules sandwiched at the dimer interface as predicted from functional studies of human CFTR [3, 18].

Another structural insight bearing evolutionary significance also emerges from these elegant cryo-EM studies. These structures virtually confirm that CFTR, albeit being the only ion channel in the ATP-binding cassette (ABC) transporter superfamily, retains not only the overall architecture of a typical ABC exporter in its TMDs, but also the highly conserved chemistry in the ATP binding pockets shared by all ABC transporters [19–22]. For ABC exporters, it is generally thought that ATP binding-induced NBD dimerization provides the “power stroke” to convert the resting inward-facing conformation to an outward-facing conformation of TMDs to release the bound cargo to the extracellular solution; subsequently, the free energy of ATP hydrolysis is used to separate the NBD dimer to reset the system so that the restored inward-facing TMDs can accept another substrate [23–25]. Gating of CFTR is believed to follow a similar scheme: ATP binding-induced NBD dimerization and hydrolysis-triggered separation of the NBD dimer are coupled respectively to the opening and closing of CFTR’s gate in TMDs ([1], cf. [26]). Thus, mutations that abolish ATP hydrolysis (e.g., E1371Q-hCFTR) can keep the gate open for minutes, resulting in a channel with an open probability (*P*_*o*_) close to unity [27, 28]. This strategy of stabilizing NBDs in a dimerized form to acquire the open channel structure of CFTR was adopted by Zhang et al. (2017) (3) in their latest ground-breaking cryo-EM work on zCFTR. Although the solved structure of zCFTR with the E1372Q mutation indeed shows practically prototypical NBDs in a dimerized form, the TMDs unexpectedly assume a nonconductive conformation [3]. The authors proposed that this nonconductive state likely represents a “post-open” closed state that is embedded within an open burst. To explain the conflicting fact that this closed state must be quite stable to become the dominant conformation in their EM sample preparation, but at the same time “invisible” in the human CFTR electrophysiological data, the authors further speculated that high frequency transitions between the open state and this presumed “intra-burst” closed states in human CFTR were mostly filtered out due to a limited bandwidth of recording. However, a recent molecular dynamics study suggests that this closed conformation is fairly stable during 1 μs simulation [29]. Of note, so far all functional interpretations of zCFTR structures were based on data collected from electrophysiological studies of hCFTR. A high degree of structural similarities between hCFTR and zCFTR does not guarantee that these two orthologs must behave similarly. Indeed, despite as high as 76% homology between human and mouse CFTR [30, 31], their gating behaviors and responses to some pharmacological reagents differ drastically [16, 32–39]. Thus, our mechanistic understanding of this important zCFTR structure with dimerized NBDs as well as future exploitations of the cryo-EM CFTR structures at large hinges on an urgent need for functional data with zCFTR.

In the current study, we characterized zCFTR expressed in CHO cells with the patch-clamp technique. We demonstrated that similar to WT-hCFTR, WT-zCFTR is activated by PKA-dependent phosphorylation. Phosphorylated WT-zCFTR is gated by ATP with a similar sensitivity to ATP as WT-hCFTR. At the single-channel level, however, WT-zCFTR assumes a very low *P*_*o*_ (0.03± 0.02; cf. ~0.50 for hCFTR in [40]) with exceptionally long closed events that can last for seconds, short-lived openings and frequent irregular sub-conductance states. Introducing the E1372Q mutation to abolish ATP hydrolysis indeed greatly stabilized the open burst as measured by macroscopic current decay upon removal of ATP, but contrary to hCFTR under the same condition (Po > 0.94 in [41]), abundant long closures are visible within a “locked-open” burst of E1372Q-zCFTR resulting in a *P*_*o*_ of ~0.35. The observation that the closed state, even when the NBDs are in a dimerized form in E1372Q-zCFTR, is more stable than the open state, provides a reasonable explanation for the dominance of observed closed channel conformations in the cryo-EM preparations of E1372Q-zCFTR. In summary, the current studies demonstrate that despite a high degree of structural similarities between human and zebrafish CFTR, their functional properties differ enormously including different pharmacological properties, and underscore the necessity of combining high-resolution CFTR structures and functional data in gaining insights into the molecular mechanism of CFTR gating.

## Materials and methods

### Mutagenesis and channel expression

Codon optimized zebrafish CFTR (zCFTR) was provided by Dr. Jue Chen (Rockefeller University). The native form of zebrafish CFTR (nzCFTR) was a gift from Dr. Michel Bagnat‘s lab at Duke University. Site-directed mutagenesis was done by PCR mutagenesis using the Pfu Ultra II (Agilent Technologies). All constructs were confirmed by DNA sequencing (DNA core; University of Missouri-Columbia) and amplified using Invitrogen Plasmid Miniprep Kit. Chinese hamster ovary (CHO) cell line from American Type Culture Collection, Manassas, VA, USA) was grown at 37°C and 95% O_2_−5% CO_2_ in Dulbecco’s modified Eagle’s medium (Life Technologies, Inc., Rockville, MD, USA) containing 10% fetal bovine serum (Harlan Biosciences, Madison, WI, USA). The cDNA constructs of CFTR were co-transfected with peGFP-C3 (Takara Bio Inc.) encoding the green fluorescent protein using PolyFect transfection reagent (QIAGEN) into CHO cells. The transfected cells were transferred into 35 mm tissue culture dishes containing one layer of sterilized glass chips for cells to grow on. The transfected cells were incubated at 27°C for 2–7 days before experiments were performed.

### Reagents and electrophysiology

Most of patch-clamp experiments were carried out in the excised inside-out configuration. Micropipettes made of borosilicate capillary glass were pulled with a two-stage vertical puller (Narishige) and then fire-polished with a homemade microforge to reach a pipet resistance of 2−7 MΩ when the pipettes were filled with a standard inside-out pipet solution that contains: 140 mM N-Methyl-D-glucamine (NMDG)-Cl, 2 mM MgCl_2_, 5 mM CaCl_2_ and 10 mM HEPES, adjusted to pH 7.4 with NMDG. A glass chip with transfected cells grown on was placed into a chamber on the stage of an inverted microscope (Olympus) and continuously perfused with a bath solution (145 mM NaCl, 5 mM KCl, 2 mM MgCl_2_, 1 mM CaCl_2_, 5 mM glucose, 5 mM HEPES, and 20 mM sucrose, adjusted to pH 7.4 with NaOH). Immediately after a membrane patch reached a seal resistance of > 40 GΩ, it was excised and continuously perfused with a standard perfusate (150 mM NMDG-Cl, 10 mM EGTA, 10 mM HEPES, 8 mM Tris, and 2 mM MgCl_2_, adjusted to pH 7.4 with NMDG).

Experiments were conducted at room temperature (22–24°C). Current signals were acquired with a patch-clamp amplifier (EPC9, HEKA), filtered at 100 Hz, digitized online at 500 Hz with Pulse software (version 8.53, HEKA) and captured onto a hard disk. Fast solution exchange was achieved with a commercial solution exchange system (SF-77B Perfusion Fast-Step, Warner Instruments).

GlyH-101 blocking data were carried out in the whole-cell configuration using micropipettes made as described above with a resistance of 1.5–2.5 MΩ when filled with whole-cell pipet solution (10 mM EGTA, 10 mM HEPES, 20 mM TEACl, 10 mM MgATP, 2 mM MgCl_2_, 101 mM CsCl, and 5.8 mM glucose, with pH adjusted to 7.4 using CsOH). During whole-cell recording, the cell was perfused with the same bath solution as the one used for inside-out recordings. Furthermore, solutions used in the whole-cell recordings (forskolin and GlyH-101) were made with the same bath solution. Whole-cell currents were recorded with a 200 ms voltage ramp of ± 100 mV applied every 5 s. The signals were acquired with a patch-clamp amplifier (EPC9, HEKA), filtered at 1 kHz with an eight-pole Bessel filter (LPF-8, Warner Instruments), digitized online at 2 kHz with Pulse software (version 8.53, HEKA), and captured onto a hard disk.

**Fig 1:**
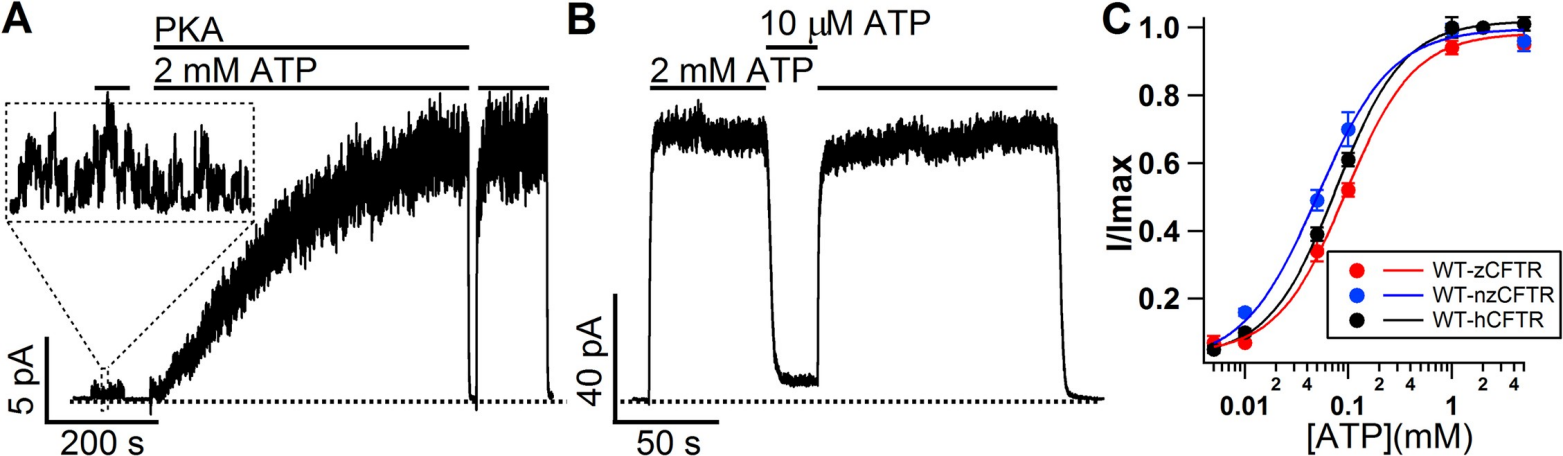
zCFTR is a phosphorylation-activated and ATP-gated channel with similar sensitivity to ATP compared with hCFTR. **(A)** Real-time macroscopic current trace showing PKA phosphorylation-dependent activation and ATP-dependent gating of WT-zCFTR. Right after patch excision, the application of 2 mM ATP only elicits minimal amount of channel activity (expanded in dashed square) which is eliminated completely upon ATP washout. The application of PKA plus 2 mM ATP robustly increases the macroscopic current to a steady-state. After the current declines to the baseline upon washing out the PKA/ATP cocktail, reapplication of 2 mM ATP alone is able to reopen the channels to the same level of activity as PKA and ATP combined, indicating a full phosphorylation of zCFTR with negligible dephosphorylation by membrane-associated phosphatases. **(B)** A representative macroscopic current trace showing the [ATP]-dependence of phosphorylated WT-zCFTR. The macroscopic current level in the presence of 10 μM ATP is only about 7% of that at 2 mM ATP. Dashed lines in A and B mark the closed-channel current level. **(C)** Normalized ATP dose-response relationships for codon optimized zCFTR (WT-zCFTR), native form zCFTR (WT-nzCFTR), and WT-hCFTR. Solid lines (red: WT-zCFTR, blue: WT-nzCFTR, black: WT-hCFTR) represent fits of the data with the Hill equation. Macroscopic CFTR current values at different concentrations of ATP were normalized to that in the presence of 2 mM ATP. The K_1/2_ for codon optimized zCFTR (WT-zCFTR) and native form zCFTR (WT-nzCFTR) are 0.09 mM (Hill coefficient = 1.3) and 0.05 mM (Hill coefficient = 1.2) respectively. The K_1/2_ for WT-hCFTR data is 0.076 mM (Hill coefficient = 1.3 with data from Zhou et al., 2006). For each data point, N = 3–10.

ATP (SigmaAldrich) containing solutions were made with the standard perfusate to different concentrations as indicated in Fig 1 for ATP dose-response measurement. To keep the reducing environment for PKA (SigmaAldrich), we routinely added 2.67 mM dithiothreitol (DTT) (SigmaAldrich) to the standard perfusate containing 32 IU/mL PKA and 2 mM ATP. 2′‐deoxy‐ATP (dATP), and pyrophosphate (PP_i_) was purchased from Sigma-Aldrich Co. LLC. *N* ^6^‐Phenylethyl‐ATP (P‐ATP) was purchased from Biology Life Science Institute (Bremen, Germany). GlyH-101 and CFTR_inh_-172 (Inh-172) were kindly provided by Dr. Robert Bridges (Department of Physiology and Biophysics, Rosalind Franklin University, Chicago, IL) with support from the Cystic Fibrosis Foundation Therapeutics. Forskolin was purchased from Enzo Life Sciences, Inc.

### Data analysis

**Fig 2:**
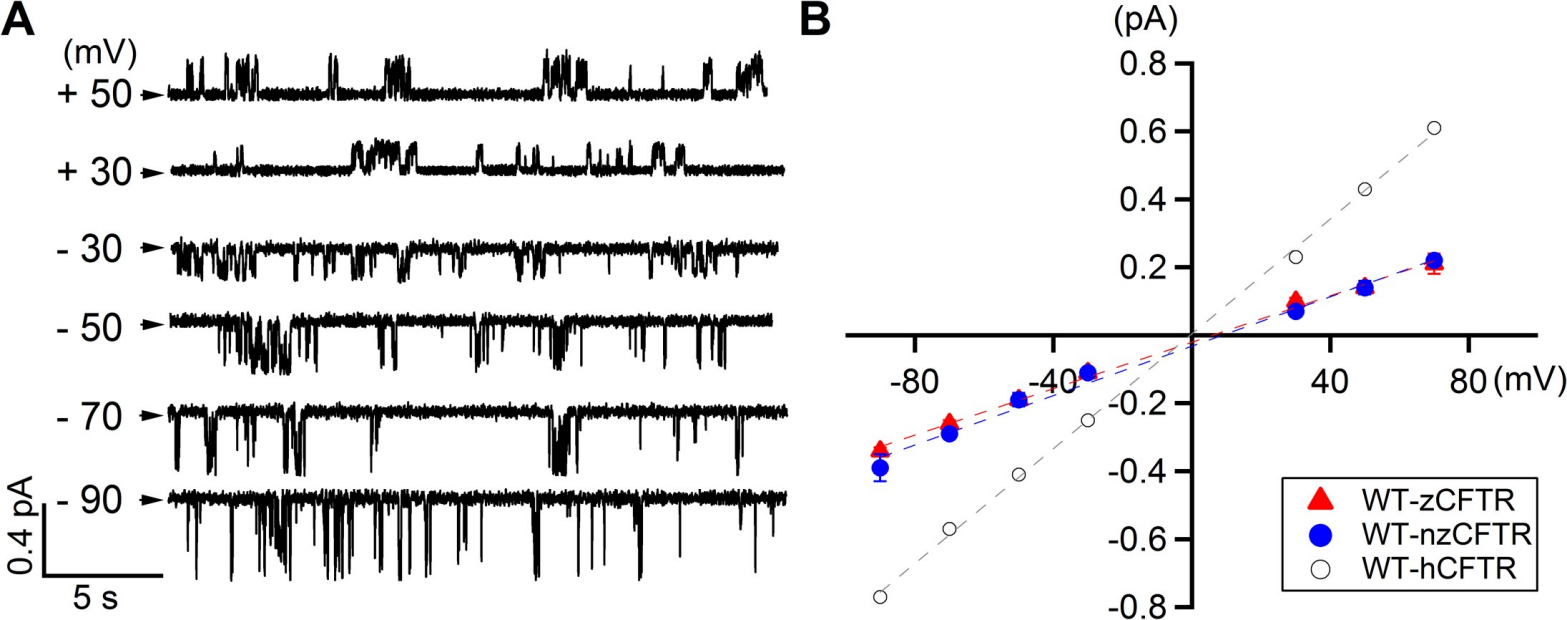
Single-channel conductance of phosphorylated zCFTR. **(A)** Single channel recordings of WT-zCFTR recorded at different membrane potentials from + 50 mV to—90 mV. In addition to smaller channel conductance, multiple sub-conductance levels were often observed. Arrowheads indicate the closed-channel current level. **(B)** Single-channel I-V relationship of zCFTR shows smaller conductance and inward-rectification. The single-channel I-V relationship of WT-hCFTR [41] is shown in circles for comparison. The I-V relationships of both codon optimized zCFTR (WT-zCFTR, red triangle) and native form zCFTR (WT-nzCFTR, blue dot) are nearly identical. Linear-fittings (dashed lines) of the I-V relationship show that the overall conductance is 8.4 pS for WT-hCFTR, 3.4 pS for WT-zCFTR and 3.6 pS for WT-nzCFTR. For each data point, N = 3–10.

Current traces recorded at negative voltage were presented in all figures as upward deflections for the sake of clear presentation except for those in Fig 2A, where positive currents were presented as upward deflections. The single-channel amplitudes were measured by fitting the distributions in all-points histograms with built-in multi-Gaussian function (Igor 6.3). In addition, in the all-points histograms, the model-independent *P*_*o*_ was calculated by dividing the area under open curve by the overall areas under both the open and closed curves with the midpoint of the two peaks as the cutoff to define open events. Significant difference was calculated through a two-sample t-test (two-tailed) using Minitab 18 (Minitab Inc.) and p < 0.05 was considered significant. Single-channel dwell-time analysis was conducted using a home-made program [28, 42] with the same cutoff to define open events as used in the all-points histograms. All results are presented as means ± SEM; N is the number of experiments.

## Results

To characterize the functional properties of zCFTR, we expressed zCFTR in CHO cells and recorded the current in inside-out patches. Not surprisingly, zCFTR is a PKA-activated, but ATP-gated chloride channel. Fig 1A shows a real-time recording of zCFTR channel currents: Just like its human counterpart [43, 44], very small currents were elicited by 2 mM ATP alone prior to the addition of PKA (expanded in the dashed box). The application of PKA and ATP cocktail dramatically increased the currents which dissipated in seconds once the PKA and ATP cocktail was washed out, but a reapplication of ATP without PKA promptly restored the currents. The fact that application of ATP alone without PKA can reach the current level as that in the presence of PKA and ATP cocktail suggests an absence of significant dephosphorylation in our excised patch system and thus a full phosphorylation of zCFTR channels. This ATP-dependent gating of phosphorylated zCFTR was reaffirmed in Fig 1B showing that the currents of phosphorylated zCFTR in the presence of 10 μM ATP are only about 7% of that at 2 mM ATP. Plotting the fraction of currents from phosphorylated zCFTR channels at different [ATP], we obtained normalized [ATP] dose response relationships for both the codon optimized (WT-zCFTR) and the native form of zCFTR (WT-nzCFTR). Compared to that of WT-hCFTR [45], the sensitivities of both zCFTR constructs to changes of [ATP] are virtually the same (Fig 1C).

Given the macroscopic data presented in Fig 1, it seems that zCFTR is functionally similar to hCFTR. However, the single-channel recordings of zCFTR reveal immensely different functional properties. First, unlike hCFTR with clear, readily discernible opening and closing events that last for tens to hundreds of milliseconds, the single-channel recording of zCFTR shows very different gating patterns (more details in S1 Fig). In addition, compared to hCFTR, the single-channel conductance of zCFTR is smaller and shows multiple sub-conductance levels, which are difficult to resolve because of their brief durations. Fig 2A presents single-channel traces of zCFTR at different holding potentials. Moreover, the single-channel I-V curves of zCFTR (both codon-optimized and native form) in Fig 2B show a clear inward-rectification. Thus, the observed smaller single-channel amplitudes and the inward-rectification suggest that the TMDs especially the pore architecture of the open channel of zCFTR should bear some structural differences from that of hCFTR.

**Fig 3:**
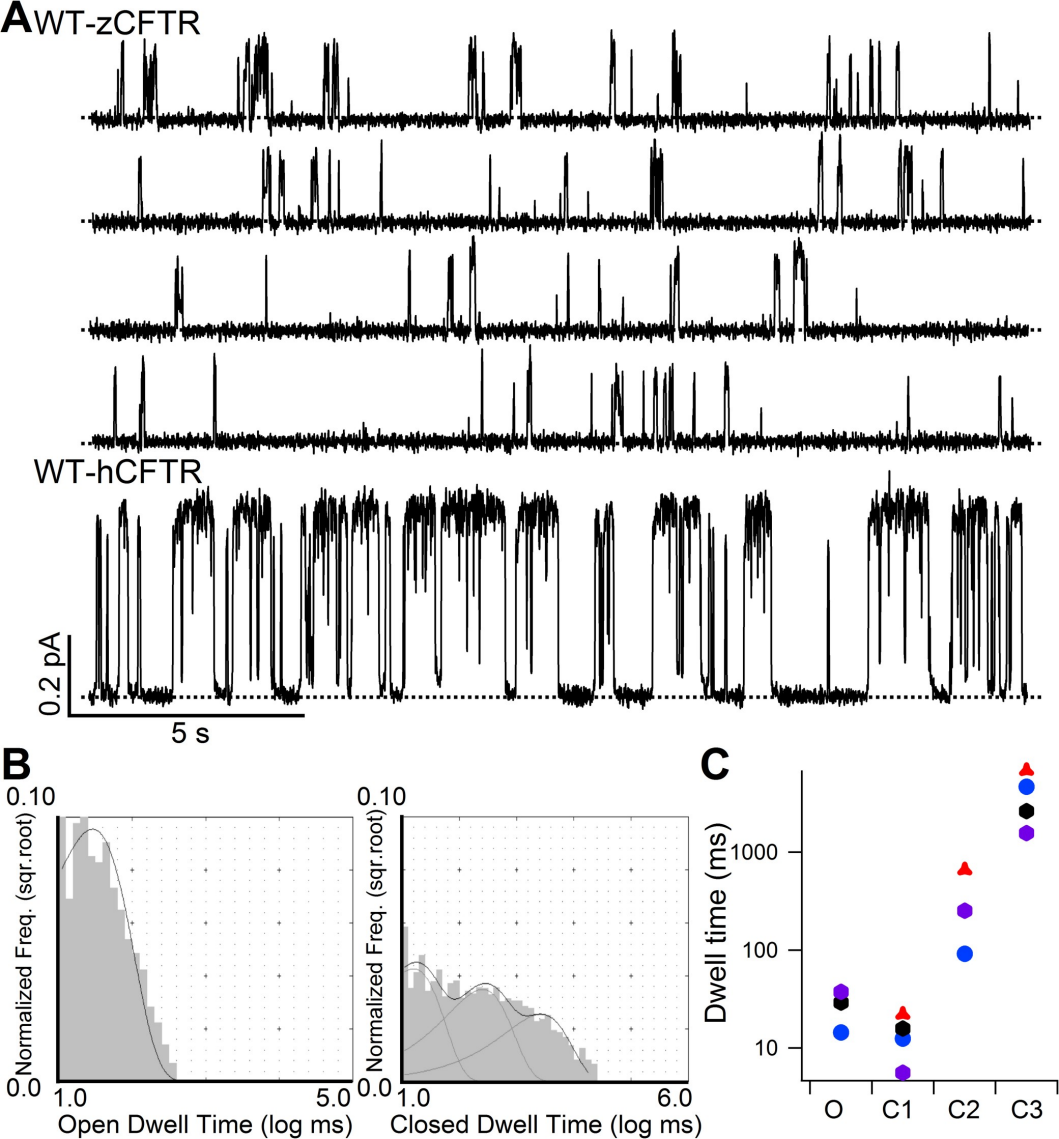
Single-channel behavior and dwell-time analysis of WT-zCFTR. **(A)** Continuous single-channel recording of WT-zCFTR at -50 mV showing brief openings and long closures, in stark contrast to well defined opening bursts and interburst closures seen in WT-hCFTR. Dashed lines mark the closed-channel current level. **(B)** Dwell-time analysis of WT-zCFTR resolved one single component for the opening events and three populations for closures. **(C)** Time constants extracted from the analysis shown in panel B reveal two stable closed states (time constants: τ_C2_ = 314 ± 122 ms N = 4, τ_C3_ = 3933 ± 1167 ms N = 4, respectively) in addition to a short-lived closure (τ_C1_ = 14 ± 4 ms, N = 4) while the opening time constant is very small (τ_O_ = 24 ± 6 ms, N = 4). Four different colors were used to represent four separate recordings.

Fig 3A shows an 80-s real-time continuous single-channel recording of phosphorylated WT-zCFTR in the presence of 2 mM ATP. A short segment of single-channel recording of WT-hCFTR with the same time and conductance scale is displayed for comparison. Unlike WT-hCFTR, the opening events of WT-zCFTR are mostly very brief; some of the openings are so brief that the full opening is not resolved because of filtering. While the bursting behavior of WT-hCFTR has been well described (hundreds of milliseconds bursts with short-lived intra-burst closure of ~5 ms in duration in [41]), it is difficult to define a burst for WT-zCFTR. In addition, many closed events that last longer than 1 s were easily seen. Thus, the *P*_*o*_ of zCFTR must be much lower than that of WT-hCFTR. In light of this unusual gating behavior of WT-zCFTR, we first generated all-points histograms, which yields a model-independent *P*_*o*_ of 0.03 ± 0.02 (n = 4), 15-fold lower than that of WT-hCFTR [40]. Subsequently, we used single-channel opening and closing events in the presence of 2 mM ATP to construct dwell-time histograms (Fig 3B): While the open time histogram can be well fitted with a single exponential function (τ_O_ = 24 ± 6 ms, N = 4), three populations of the closed events are observed (τ_C1_ = 14 ± 4 ms, τ_C2_ = 314 ± 122 ms, τ_C3_ = 3933 ± 1167 ms, N = 4). In a latest report, we employed similar analysis for hCFTR and obtained τ_O_ = 200 ms and τ_C1_ = 5 ms and τ_C2_ = 502 ms [41]. Fig 3C summarizes results from this dwell-time analysis from four different patches. Although we observed significant variations, it is safe to conclude that zCFTR strongly prefers to stay in the closed states even in the presence of saturating [ATP]. Collectively, the data in Figs 1 and 3 suggest that although WT-zCFTR and WT-hCFTR show similar [ATP] sensitivities, inspection of the raw trace of WT-zCFTR reveals that most opening events are brief as if once the channel opens, the open state is not stable and may transition to a fairly stable “intraburst” closed state (see Discussion). This impression was further confirmed by recording of a mutant zCFTR with the catalytic glutamate neutralized (see below).

**Fig 4:**
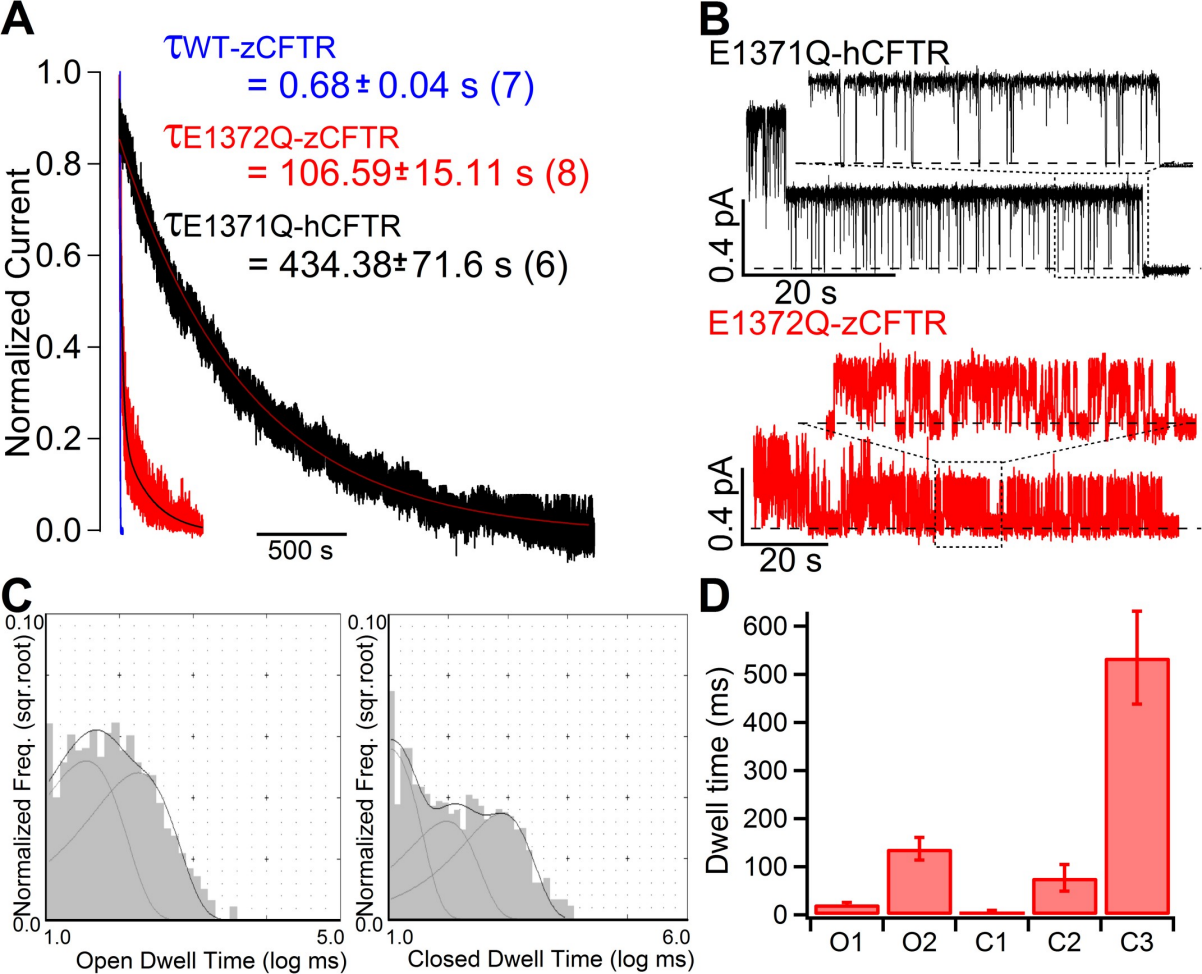
Effects of ATP hydrolysis on zCFTR gating. **(A)** Normalized current relaxations upon ATP removal for WT-zCFTR (blue), E1372Q-zCFTR (red) and E1371Q-hCFTR (black). Steady-state macroscopic mean currents at time 0 when ATP was removed were normalized as 1 and the fitted curves are superimposed with decays of the raw current traces. The relaxation time constant for each construct is presented. A > 100-fold increase of the relaxation time constant by the E1372Q mutation in zCFTR suggests that ATP hydrolysis plays a role in zCFTR gating. Of note, the time constant for E1372Q-zCFTR is much smaller than that for E1371Q-hCFTR (adopted from [41]). **(B)** Representative microscopic current traces of E1371Q-hCFTR (adopted from [41]) and E1372Q-zCFTR after ATP is completely removed from the perfusate to show the gating behavior within a prolonged opening burst. Part of the traces (in dashed boxed) was expanded to show more detailed differences in single-channel behavior between E1371Q-hCFTR and E1372Q-zCFTR. Heavy dashed lines mark the closed-channel current level. **(C)** Representative dwell-time histograms for E1372Q-zCFTR suggest two open time constants and three closed time constants. **(D)** Kinetic parameters extracted from dwell-time analysis on 5 patches: τ_O1_: 22 ± 4 ms and τ_O2_: 137 ± 23 ms; τ_C1_: 6 ± 3 ms, τ_C2_: 77 ± 28 ms, τ_C3_: 535 ± 97 ms, N = 5.

Despite the drastic differences in gating kinetics between WT-hCFTR and WT-zCFTR, ATP hydrolysis does play a similar role in controlling CFTR gating for these two orthologs. Fig 4A shows a dramatic prolongation of the current relaxation upon removal of ATP by introducing the hydrolysis-deficient mutation (E1372Q) into zCFTR. However, we also noted a quantitative difference in the current relaxation time constants between human and zebrafish constructs (107 ± 15 s, N = 8 in E1372Q-zCFTR vs. 434 ± 72 s, N = 6 in E1371Q-hCFTR adopted from [41], p < 0.005). More striking differences reside in the detailed gating kinetics in a locked-open burst of E1372Q-zCFTR (Fig 4B). Different from a *P*_*o*_ close to unity within the locked-open burst of E1371Q-hCFTR, E1372Q-zCFTR shows more frequent and longer closures within one locked-open burst. Quantitatively, a model-independent *P*_*o*_ analysis within the locked-open burst generated with all-points histograms yields a *P*_*o*_ of 0.35 ± 0.05, (N = 9). Since a locked-open burst is considered a state with a stabilized NBD dimer, this latter observation suggests that E1372Q-zCFTR prefers closed conformations even with stably dimerized NBDs. Fig 4C shows representative dwell-time histograms with the open time histogram best fitted with two components and the closed time histogram with three components (Fig 4D). It is worth noting that this low *P*_*o*_ is not due to previously proposed voltage-dependent blockade of the pore by small molecules in the cytoplasmic solution [46–48], since similar frequent intra-burst closures are also observed in recordings at a positive voltage (S2 Fig). Collectively, the preference for closed channel conformations in E1372Q-zCFTR over open states offers a simple explanation for why a non-conductive state with dimerized NBDs is captured as the dominant conformation in the cryo-EM study [3]. The drastic differences in gating behavior between hCFTR and zCFTR with ATP hydrolysis abolished or not also highlight the limitation of interpreting the structures of zCFTR with only functional data of hCFTR.

**Fig 5:**
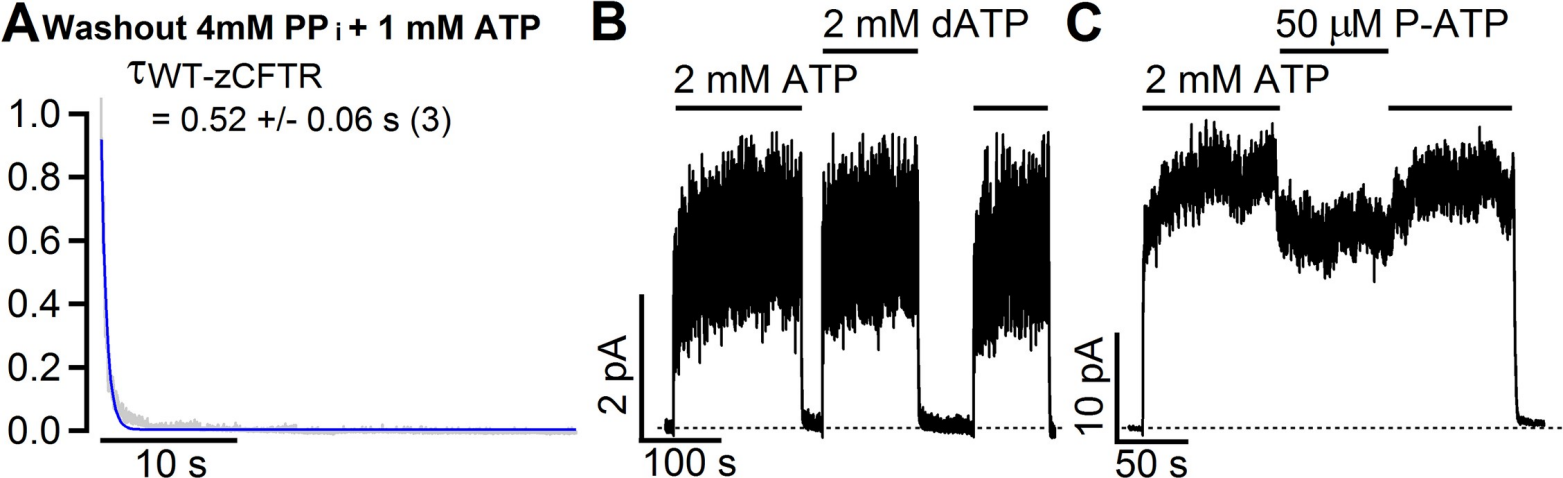
zCFTR shows different responses to ATP and phosphate analogues. **(A)** Normalized current decay upon washing out 1 mM ATP and 4 mM MgPP_i_ shows almost unaltered relaxation time constant (0.52 ± 0.06 s, N = 3) as compared with washing out 2 mM ATP (0.68 s in Fig 4A) for WT-zCFTR. Similarly, removal of 1 mM ATP and 4 mM MgPP_i_ on WT-nzCFTR yields a time constant of 0.58 ± 0.02 s (N = 3). In contrast, the same experiments on WT-hCFTR yield a current decay time constant of 31.3 ± 3.2 s (N = 8). **(B)** A real time recording showing that 2 mM dATP elicits a similar macroscopic current as 2 mM ATP for zCFTR. Of note, a previous report showed an increase of *P*_*o*_ from 0.45 (2 mM ATP) to 0.82 (2 mM dATP) for human WT-CFTR [51]. **(C)** A real time recording of WT-zCFTR shows a slight decrease of the macroscopic current upon switching the ligand from 2 mM ATP to 50 μM P-ATP. In contrast, switching from 2.75 mM ATP to 50 μM P-ATP results in ~ 2-fold current increase in hCFTR [49].

To further investigate the NBD machinery of zCFTR, we tested the effect of magnesium pyrophosphate (Mg^2+^PP_i_) and ATP analogues dATP and P-ATP. First, Mg^2+^PP_i_ is reported to effectively lock-open hCFTR once applied together with lower concentration of ATP [49, 50]. Thus as a result, the time constant of the current decay upon washing out the cocktail (1 mM Mg^2+^ATP and 4 mM Mg^2+^PP_i_) is dramatically prolonged to 31.3 ± 3.2 s (N = 8) [49]. In our study, the application of the same cocktail (1 mM Mg^2+^ATP and 4 mM Mg^2+^PP_i_) is not able to lock open the channels since the time constant from single-exponential fitting of the current decay phase upon washing out the cocktail (Fig 5A) is almost identical to that upon washing out 2 mM ATP alone (Fig 4A). Second, a previous report showed that 2 mM dATP can increase the *P*_*o*_ WT-hCFTR from 0.45 (2 mM ATP) to 0.82 (2 mM dATP) [51]. However, the representative recording in Fig 5B shows that the same concentration of 2 mM dATP yields an overall similar channel activity as 2 mM ATP for zebrafish CFTR. The fold increase of macroscopic mean current (I_2 mM dATP/I_2 mM ATP) is 1.01± 0.01 (N = 5) and 1.00 ± 0.07 (N = 7) for WT-zCFTR and WT-nzCFTR, respectively. Third, a real time recording of WT-zCFTR in Fig 5C shows a slight decrease of the macroscopic current upon switching the ligand from 2 mM ATP to 50 μM P-ATP (I_P-ATP_/I_ATP_ = 0.88 ± 0.04, N = 5, for WT-zCFTR; I_P-ATP_/I_ATP_ = 0.85 ± 0.01, N = 4, for WT-nzCFTR). In contrast, switching from 2.75 mM ATP to 50 μM P-ATP results in ~ 2-fold current increase in hCFTR [49]. Collectively, the zCFTR shows different responses to ATP and phosphate analogues despite the highly conserved NBDs between zCFTR and hCFTR.

**Fig 6:**
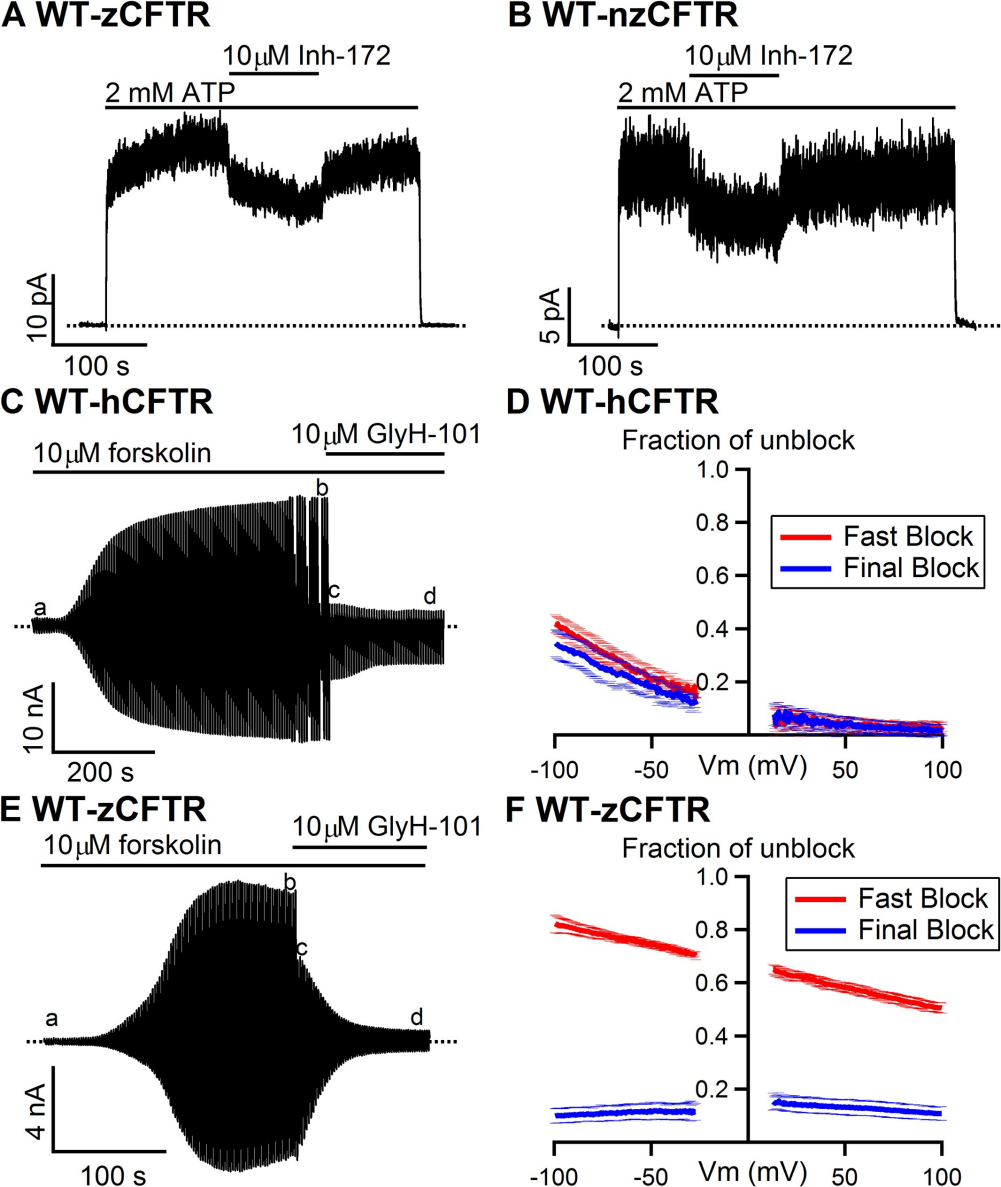
Different pharmacological responses to CFTR inhibitor/blocker between zebrafish and human CFTR. **(A)** and **(B)** Whereas 10 μM CFTR_inh_-172 was reported to eliminate about 90% of WT-hCFTR current [52], the same concentration of the reagent shows less inhibitory effect on WT-zCFTR. The virtually identical properties of both WT-zCFTR and WT-nzCFTR were confirmed at multiple points in the current study (e.g., Figs 1 and 2) to avoid biased interpretations of our functional data because of potential changes in structure and function due to different protein translation rates caused by codon manipulations reported previously [54]. **(C)** A representative whole-cell recording of WT-hCFTR in response to external application of 10 μM GlyH-101. In whole-cell recordings, right after break-in (a), the application 10 **μ**M forskolin elicits large CFTR current (b). A biphasic current drop upon the application of GlyH-101 was observed: an immediate decrease of the current (c) followed by a slow phase of current decrease (d). **(D)** Averaged fractions of unblock for both the fast phase (red) and the eventual steady-state (blue) from three recordings show voltage-dependence of external GlyH-101 block on WT-hCFTR. **(E)** A representative whole-cell recording of WT-zCFTR upon external application of 10 **μ**M GlyH-101. In addition to an abrupt current drop, a slow current decay was also observed. **(F)** Averaged fractions of unblock for both the fast phase (red) and the final block (blue) from three recordings show voltage-dependence of the fast block and voltage-independence of the final block on WT-zCFTR.

We next examined if zCFTR and hCFTR respond similarly to two specific CFTR inhibitors CFTR_inh_-172 (Inh-172) and GlyH-101. While 10 μM Inh-172 only inhibits ~ 30% of the current for zCFTR (inhibition ratio: 0.27 ± 0.03, N = 5 for WT-zCFTR and 0.30 ± 0.04, N = 4 for WT-nzCFTR in Fig 6A and 6B) in inside-out patches, the same concentration of Inh-172 inhibits the macroscopic current of WT-hCFTR by ~ 90% [52]. Moreover, whole-cell recordings also reveal different responses of WT-hCFTR (Fig 6C and 6D) and WT-zCFTR (Fig 6E and 6F) to the external application of 10 μM GlyH-101. Forskolin-induced whole-cell CFTR currents (compare a and b in Fig 6C and 6E) decrease upon exposure to external GlyH-101 in two distinct phases. A majority of the hCFTR currents were blocked in the fast phase (compare b and c in Fig 6C), which is followed by a minor fraction of slow phase blockade (compare c and d in Fig 6C). For zCFTR, although the fast phase block (compare b and c in Fig 6E) exhibits a clear voltage-dependence like the one seen in hCFTR (red lines in Fig 6D and 6F), the overall magnitude of block is much smaller. However, different from hCFTR, the final steady-state block in zCFTR is larger and voltage-independent (compare blue lines in Fig 6D and 6F). The biphasic current decay suggests either two blocking processes or two GlyH-101 binding sites in WT-zCFTR. While we do not know the underlining mechanism for the slow block, it is safe to assume that the fast-phase voltage-dependent block represents plugging of the external pore of CFTR as proposed by Norimatsu et al. [53]. In that context, hCFTR appears to be more sensitive to GlyH-101 than zCFTR.

## Discussion

In the current study, we characterized the electrophysiological properties of zCFTR and found gating and ion-conducting features of zCFTR distinct from its human brother, although zCFTR channel, like its human ortholog, is activated by PKA-dependent phosphorylation and gated by ATP. Notwithstanding the remarkable similarities between hCFTR and zCFTR in their apo structures [1, 4], the single-channel conductance of zCFTR is smaller than that of hCFTR with a clear inward rectification (Fig 2B), suggesting the open states of these two constructs bear structural differences in their respective anion permeation pathways. Interestingly, most of the positively charged amino acids lining the internal vestibule of hCFTR, such as K95, E92, K190, R248 (K in zCFTR), R251 (K in zCFTR), R303, R352, D363, K370, D993, K1041 and R1048 are conserved in zCFTR [48, 55–61], suggesting that a mechanism more complicated than simple electrostatics imposed by these positive charges accounts for this difference in ion permeation. More strikingly, despite a nearly identical sensitivity to ATP, gating of phosphorylated zCFTR at a saturating [ATP] is greatly subdued with an open probability more than 15-fold lower than that of hCFTR (*P*_*o*_ = 0.03 for zCFTR in Fig 3 versus *P*_*o*_ = 0.50 for hCFTR in [40]). Detailed kinetic analysis suggests that this low *P*_*o*_ is mostly due to brief openings and long closings.

Specifically, our dwell-time analysis of the single-channel recordings of WT-zCFTR revealed one open time constant and three closed time constants: (τ_O_ = 24 ± 6 ms, N = 4; τ_C1_ = 14 ± 4 ms, τ_C2_ = 314 ± 122 ms, τ_C3_ = 3933 ± 1167 ms, N = 4), different from those reported previously for hCFTR (τ_O_ = 200 ms and τ_C1_ = 5 ms and τ_C2_ = 502 ms in [41]). To eliminate the brief closures that are traditionally considered as events not related to ATP-dependent gating [46, 47, 62], we assumed the shortest closed state component τ_C1_ (= 14 ± 4 ms, N = 4) represents this ATP-independent closing and carried out burst analysis by setting a delimiter (79 ms) between the first two peaks (τ_C1_ and τ_C2_) of the closed time histogram, and obtained an open burst duration 66 ± 23 ms (N = 4) and two long closed time constants (612 ± 219 ms N = 4 and 5096 ± 1834 ms N = 4, respectively). Of note, same analysis of WT-hCFTR yielded τ_burst_ = 386 ms and τ_interburst_ = 480 ms (reported in [41]). We noted that for WT-hCFTR, the burst duration estimated from microscopic analysis (386 ms) is very similar to the macroscopic current relaxation time constant (~460 ms in [63]), but there is a major discrepancy for WT-zCFTR (66 ms versus 680 ms in Fig 4A). As macroscopic relaxation analysis, unlike microscopic burst analysis, is less arbitrary, a relaxation time constant of 680 ms suggests that the real burst duration for WT-zCFTR is much longer than 66 ms. Taking this scenario into consideration upon examining the single-channel current trace (Fig 3), we conclude that not only does WT-zCFTR stay closed for hundreds of milliseconds to seconds without sojourning to an open burst state, the open channel is also very unstable and transits to more stable closed conformation(s) repeatedly in an open burst. This latter proposition is supported by the single-channel data of E1372Q-zCFTR (Fig 4) where closed events lasting for hundreds of milliseconds can be readily discernible in a “locked-open” burst. If τ_C2_ of WT-zCFTR indeed represents one of these stable closed conformations buried within an open burst, which are not traditionally seen in the opening bursts of WT-hCFTR, a better burst analysis for zCFTR is to set the delimiter between τ_C2_ and τ_C3_ (1585 ms). Two open burst states and one interburst state were resolved (τ_Ob1_ = 25.1 ± 4.0 ms, τ_Ob2_ = 1478 ± 494 ms and τ_ib_ = 4083 ± 1037 ms, N = 4) with the probabilities of Ob1 and Ob2 events as 0.48 ± 0.09 and 0.52 ± 0.09, respectively (N = 4). This alternative analysis revealed a long open burst population whose time constant (τ_Ob2_ = 1478 ± 494 ms) fits better the ATP-dependent “open” burst duration of WT-zCFTR, which is normally measured by the macroscopic current relaxation (680 ms in Fig 4A). Although this alternative analysis did not completely resolve the discrepancy given the limited number of single-channel patches and the large variance of τ_Ob2_ and τ_ib_, without referring to any gating model, we can conclude that zCFTR strongly prefers the closed channel conformation rather than the open state even in the presence of millimolar ATP that is sufficient to saturate the binding at NBDs (Fig 1C).

Like its human ortholog, zCFTR retains the capacity of using ATP hydrolysis to terminate its open bursts as mutating the catalytic glutamate (E1372) drastically prolongs the current relaxation upon removal of ATP (cf. current relaxation time constants in Fig 4A: 0.68 s versus 101 s). Most interestingly, unlike E1371Q-hCFTR whose *P*_*o*_ is close to unity [27, 28], E1372Q-zCFTR shows a much lower *P*_*o*_ of ~0.35 within a locked-open burst (Fig 4B). Here, the single-channel open bursts of E1372Q-zCFTR present closed time constants in the range of tens to hundreds of milliseconds (Fig 4B, 4C and 4D). Hence, even when ATP hydrolysis is eliminated, the closed state of zCFTR is still more stable than the open state, which echoes the findings described above for WT-zCFTR channels. Our results thus offer a simple explanation why in the cryo-EM structure of E1372Q-zCFTR, despite with a characteristic NBD dimer the segment identified previously [64] as the gate in CFTR’s ion permeation pathway is in a closed configuration. However, other possible factors including different environmental conditions such as detergent instead of membrane lipids used to solubilize CFTR and low imaging temperature could also contribute [3]. Indeed, the presence of detergent may account for the observation that in the latest cryo-EM structure of phosphorylated ATP-bound human CFTR variant (E1371Q), the aqueous pore is constricted at a narrow point [2].

As a closed state with dimerized NBDs had been considered as a transition (or unstable transient) state [12, 13], Zhang et al. (2017) proposed that the captured non-conductive conformation of E1372Q-zCFTR more likely represents a “post-open” closed state (named C_flicker_ thereafter) based on hCFTR functional data. Accordingly, the zCFTR channel visits C_flicker_ after it is first opened by ATP, which can be described by a scheme: C_long_<——>O↔C_flicker_ (cf. [3]). In this scheme, C_long_<——>O represents an ATP-dependent gating cycle that includes ATP binding, NBD dimerization, gate opening and gate closing following ATP hydrolysis and separation of the NBD dimer. It was also proposed that the transitions (O↔C_flicker_) reflect quick ATP-independent motions of parts of the TMDs (mobile external segments of TM8 and TM12) between an open and a closed conformation while the NBDs remain dimerized [3]. Since such a “post-open” C_flicker_ state, although stable enough to be captured as dominant in cryo-EM E1372Q-zCFTR structure [3], was not observed in human functional data, this “post-open” idea was further used to explain human CFTR functional data especially E1371Q-hCFTR by speculating that frequent and abundant O↔C_flicker_ transitions in E1371Q-hCFTR were filtered out because of the low bandwidth (100 Hz) of recordings, resulting in a measured *P*_*o*_ close to unity. In the current study, by demonstrating very different gating behaviors between E1372Q-zCFTR and E1371Q-hCFTR at a single-channel level, we found that the extrapolation of the idea of a “post-open” closed state based on the cryo-EM results of E1372Q-zCFTR to explain E1371Q-hCFTR functional data is unnecessary. Our data indicate that the closed state of zCFTR is simply more stable relative to the open state.

Although our functional data offer a reasonable explanation the closed state structure of E1372Q-zCFTR, a puzzle arises when we look into the published data regarding the ATP turnover rate. The measured ATP hydrolysis rate for one zebrafish CFTR protein (0.66 s^-1^) is similar to, or slightly higher than, that of human CFTR (0.37 s^-1^) [1, 4]. These biochemical data seem at odds with the differences in gating kinetics between WT-hCFTR and WT-zCFTR if we assume a one-to-one stoichiometry between the ATP hydrolysis cycle and the gating cycle. The burst analysis described above suggests that compared to WT-hCFTR, WT-zCFTR exhibits a longer burst duration (i.e., 680 ms) and much longer closed times before gate opening (interburst duration of either 5096 ms or 4083 ms). If the proposed one-to-one stoichiometry between gating cycle and ATP hydrolysis cycle is valid, the ATP hydrolysis rate for WT-hCFTR is expected to be higher than that of WT-zCFTR. While the reason for this discrepancy is unclear, one possibility is that ATP hydrolysis can occur in those prolonged closed events in WT-zCFTR. This scenario would require the existence of a closed channel conformation with dimerized NBDs as ATP hydrolysis is supposed to occur at the dimer interface when two ATP molecules are sandwiched at the dimer interface [20, 27, 65]. This same idea then places this hydrolysis-competent closed state of WT-zCFTR before gate opening (i.e., a pre-open closed state proposed in [14]). While other possibilities may exist and more studies are needed to solve this puzzle, it is worthwhile to note that the cryo-EM structure of E1372Q-zCFTR, albeit a closed state, indeed possesses a prototypical NBD dimer with almost all the conventional interactions between ATP molecules and the conserved motifs at the dimer interface especially those within the consensus ATP binding pocket [3].

In addition to the differences described above, we also found some unexpected pharmacological properties in WT-zCFTR. For instance, different from the ability of PP_i_ to lock open WT-hCFTR [50, 66, 67], the effect of PP_i_ on WT-zCFTR is negligibly small (Fig 5). More strikingly, 2’-deoxy-ATP (dATP) and P-ATP, both more effective ligands than ATP for WT-hCFTR [51, 63], did not gate WT-zCFTR to a higher level (Fig 5) under the same condition, suggesting some functional differences in NBDs and/or in the coupling between NBDs and the gate in TMDs. Of note, the cryo-EM structure of E1372Q-zCFTR shows a closed state but with the NBDs in a dimerized form. However, the same structure also shows an asymmetrical NBD dimer where the degenerate ATP binding pocket (or site 1) is not as tightly conjoined as the consensus pocket in site 2. In contrast, the latest report of phosphorylated ATP-bound hCFTR structure shows symmetrical NBD dimer (2). Thus, although the NBDs between zCFTR and hCFTR are highly conserved, the different gating behaviors of WT-zCFTR (Figs 2 and 3 and S1 Fig) and E1372Q-zCFTR (Fig 4 and S2 Fig) and the different responses of WT-zCFTR to ATP and phosphate analogues may be partly attributed to the less tight binding pocket at the degenerated pocket. While more studies are required to investigate the functional significance of this structural asymmetry in zCFTR’s NBDs, we noted that the CF-causing mutation G1349D-hCFTR in the signature sequence of site 1 shows a largely reduced *P*_*o*_ (~10-fold lower) than WT-hCFTR and is not responsive to PP_i_, likely due to the electrostatic repulsion between D1349 and the negatively charged ATP that hinders the dimerization process of the degenerated pocket [68, 69]. Thus although the NBDs are highly conserved in sequence with 66% homology between human CFTR and zebrafish CFTR, some minor structural variations in the NBDs may exist and bear significant functional consequences.

Furthermore, our data suggest detectable differences in the responses to CFTR_inh_-172 (Inh-172) and channel blocker GlyH-101 between human CFTR and zebrafish CFTR ([52] and Fig 6). Of note, previous studies have also revealed some fundamental differences in the sensitivity of CFTR to CFTR_inh_-172, glibenclamide and GlyH-101 in different species [70, 71]. For instance, shark CFTR is insensitive to CFTR_inh_-172 and pig CFTR is insensitive to Glibenclamide. Moreover, the ferret and pig airway epithelia showed less sensitivities to CFTR_inh_-172 compared with those of humans [70]. In addition, although all of human CFTR, pig CFTR and shark CFTR are responsive to GlyH-101, the inhibition ratios among species are still significantly different [71]. If we accept the idea that GlyH-101 acts as a simple pore blocker, the different responses to external GlyH-101 between WT-zCFTR and WT-hCFTR suggest structural difference in the binding sites of Gly-101 between these two orthologs. Furthermore, the apparent electrical distance (δ) of the fast-phase block from the extracellular side for WT-hCFTR is (0.49 ± 0.03, N = 4) is larger than that for WT-zCFTR (0.14 ± 0.04 N = 3) when assuming a valency of -1 and a single binding site. Thus, using GlyH-101 as a probe to explore the extracellular part of the pore, we also conclude that zCFTR and hCFTR possess different structures in the pore as implicated by the differences in the single-channel I-V relationships. As the binding site for GlyH-101 was previously proposed to reside within the external vestibule, likely in close proximity to F337 and T338 [53], these results, together with the difference in single-channel conductance (Fig 2 in main text), support the idea that the proposed narrow segment of the pore may be structurally different between zCFTR and hCFTR. Since in human CFTR this narrow segment is shown to overlap with CFTR’s gate [64], structural differences in this region could also contribute to the gating differences observed in Figs 3 and 4.

It is worth noting that previous studies on murine CFTR have already revealed diverse gating and pharmacological properties different from human CFTR. Similar to zebrafish CFTR, murine CFTR shows unstable brief openings [32]. Burst analysis of the single-channel recordings of mouse CFTR also suggests one open population and three closed populations [32]; whereas macroscopic murine CFTR current shows inward-rectified I-V relationship [37]. Moreover, PP_i_ fails to stabilize the opening of mouse CFTR [32]. Different from zebrafish and human CFTR, the murine ortholog exhibits unique opening bursts: each opening burst consists of two distinct conductance states, many brief high-conductance openings on top of a stable low conductance opening [33, 37, 72].

Collectively, our characterization of zebrafish CFTR with the patch-clamp techniques unveiled a wide spectrum of functional differences between zebrafish CFTR and its human ortholog despite their high structural similarities. The molecular mechanisms behind these surprising differences in biophysical and pharmacological properties between human and zebrafish CFTR awaits further investigations, but all the functional differences observed in the current study cannot be accounted for by the codon optimization employed for both cryo-EM and current studies since nearly identical results were obtained for native form zebrafish CFTR expressed in CHO cells. On one hand, these functional data remind us of the unrivaled temporal resolution of electrophysiological methodologies that allow monitoring the real-time dynamic properties of a single CFTR molecule in the cell membrane. On the other hand, high-resolution CFTR structures bestowed with their unparalleled spatial resolution, when put in the context of the functional data, could definitively shed light on the molecular mechanism of this medically important protein and potentially provide novel insights for the development of new therapeutic interventions in CF medicine.

## Supporting information

S1 Fig Additional representative single-channel recording of WT-zCFTR. The recording with more single-channel events recorded at -50 mV show clearly the stochastic behavior of WT-zCFTR with long closings and brief openings. Dashed lines mark the closed-channel current level (same for other figures in Supporting Information).
(DOCX)

S2 Fig E1372Q-zCFTR at +50 mV. A representative recording of E1372Q-zCFTR recorded at +50 mV after the removal of ATP shows unstable locked-open burst and suggests that the observed frequent intra-burst closures within a locked-open burst (the last one open burst after the removal of 2 mM ATP) are not due to voltage-dependent pore blocking. Note the existence of few overlapped brief opening events on top of the bursts. This is due to the fact that the patch contains many channels while the trace presents only the last locked-open channel long after ATP is removed. Thus, those brief opening events likely represent re-openings of closed channels.
(DOCX)
