# Supplementary material for: Functional characterization reveals that zebrafish CFTR prefers to occupy closed channel conformations
Source: PLoS One. 2018 Dec 31;13(12):e0209862. doi: 10.1371/journal.pone.0209862 (PMC6312236; doi:10.1371/journal.pone.0209862)
Supplement: S1 Fig — The recording with more single-channel events recorded at -50 mV show clearly the stochastic behavior of WT-zCFTR with long closings and brief openings. Dashed lines mark the closed-channel current level (same for other figures in Supporting Information). (DOCX) [file pone.0209862.s001.docx]

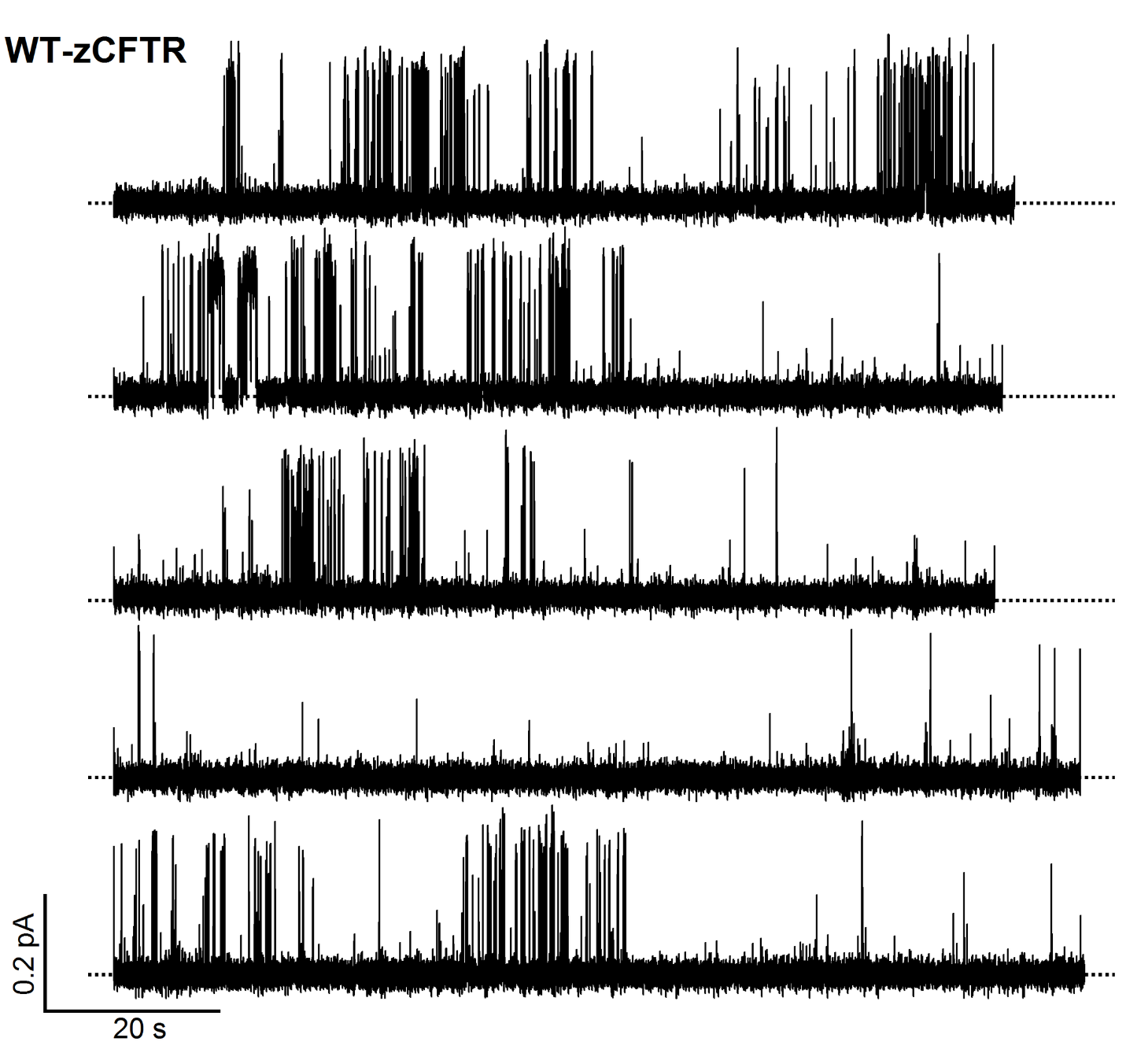


S1 Fig. Additional representative single-channel recording of WT-zCFTR. The recording with more single-channel events recorded at -50 mV show clearly the stochastic behavior of WT-zCFTR with long closings and brief openings. Dashed lines mark the closed-channel current level (same for other figures in Supporting Information).
