# Supplementary material for: Functional characterization reveals that zebrafish CFTR prefers to occupy closed channel conformations
Source: PLoS One. 2018 Dec 31;13(12):e0209862. doi: 10.1371/journal.pone.0209862 (PMC6312236; doi:10.1371/journal.pone.0209862)
Supplement: S2 Fig — A representative recording of E1372Q-zCFTR recorded at +50 mV after the removal of ATP shows unstable locked-open burst and suggests that the observed frequent intra-burst closures within a locked-open burst (the last one open burst after the removal of 2 mM ATP) are not due to voltage-dependent pore blocking. Note the existence of few overlapped brief opening events on top of the bursts. This is due to the fact that the patch contains many channels while the trace presents only the last locked-open channel long after ATP is removed. Thus, those brief opening events likely represent re-openings of closed channels. (DOCX) [file pone.0209862.s002.docx]

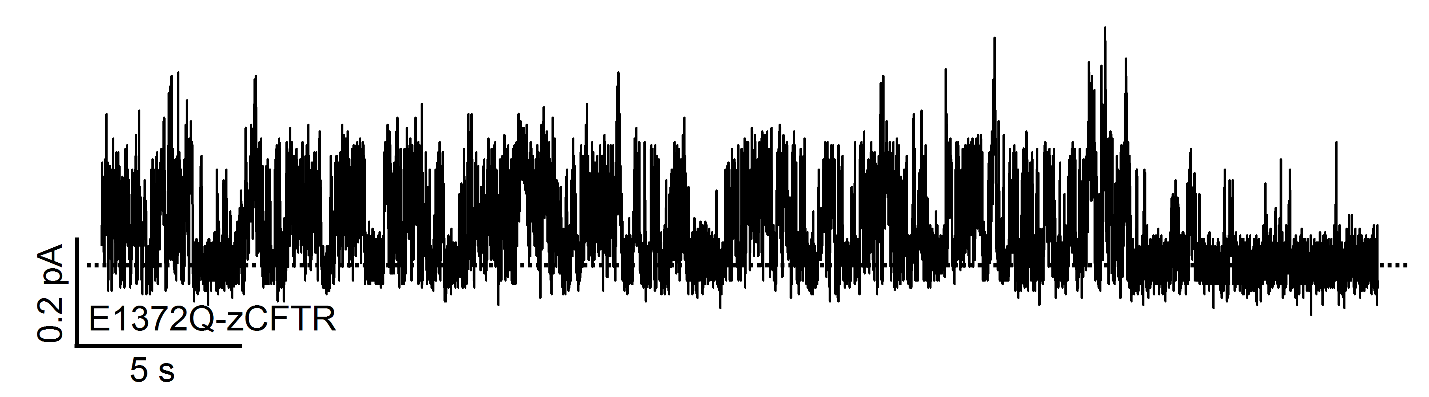


**S2 Fig. E1372Q-zCFTR at +50 mV.** A representative recording of E1372Q-zCFTR recorded at +50 mV after the removal of ATP shows unstable locked-open burst and suggests that the observed frequent intra-burst closures (Fig 4B) within a locked-open burst (the last one open burst after the removal of 2 mM ATP) are not due to voltage-dependent pore blocking [1, 2]. Note the existence of few overlapped brief opening events on top of the bursts. This is due to the fact that the patch contains many channels while the trace presents only the last locked-open channel long after ATP is removed. Thus, those brief opening events likely represent re-openings of closed channels.

1. Tabcharani JA, Linsdell P, Hanrahan JW. Halide permeation in wild-type and mutant cystic fibrosis transmembrane conductance regulator chloride channels. The Journal of general physiology. 1997;110(4):341-54. Epub 1997/10/06. PubMed PMID: 9379167; PubMed Central PMCID: PMCPMC2229372.

2. Zhou Z, Hu S, Hwang TC. Voltage-dependent flickery block of an open cystic fibrosis transmembrane conductance regulator (CFTR) channel pore. The Journal of physiology. 2001;532(Pt 2):435-48. Epub 2001/04/18. PubMed PMID: 11306662; PubMed Central PMCID: PMCPMC2278548.
